# Sex differences in blood pressure and hypertension through adulthood

**DOI:** 10.1093/ckj/sfag180

**Published:** 2026-06-04

**Authors:** Christina Thompson, Alexandre Klopp, Leonie Dreher, Dominik Kylies, Tanja Zeller, Julia Munzinger, Raphael Twerenbold, Stefan Blankenberg, Tobias B Huber, Ute Seeland, Ulrich Wenzel, Christian Schmidt-Lauber

**Affiliations:** III. Department of Medicine, University Medical Center Hamburg-Eppendorf, Hamburg, Germany; Hamburg Center for Kidney Health (HCKH), University Medical Center Hamburg-Eppendorf, Hamburg, Germany; III. Department of Medicine, University Medical Center Hamburg-Eppendorf, Hamburg, Germany; Hamburg Center for Kidney Health (HCKH), University Medical Center Hamburg-Eppendorf, Hamburg, Germany; III. Department of Medicine, University Medical Center Hamburg-Eppendorf, Hamburg, Germany; Hamburg Center for Kidney Health (HCKH), University Medical Center Hamburg-Eppendorf, Hamburg, Germany; III. Department of Medicine, University Medical Center Hamburg-Eppendorf, Hamburg, Germany; Hamburg Center for Kidney Health (HCKH), University Medical Center Hamburg-Eppendorf, Hamburg, Germany; Department of Cardiology, University Heart and Vascular Center Hamburg-Eppendorf, University Medical Center Hamburg-Eppendorf, Hamburg, Germany; Institute for Cardiogenetics, University Hospital Schleswig-Holstein, University of Lübeck, Lübeck, Germany; German Center for Cardiovascular Research (DZHK) Partner Site Hamburg-Kiel-Lübeck, Lübeck, Germany; Department of Cardiology, University Heart and Vascular Center Hamburg-Eppendorf, University Medical Center Hamburg-Eppendorf, Hamburg, Germany; Department of Cardiology, University Heart and Vascular Center Hamburg-Eppendorf, University Medical Center Hamburg-Eppendorf, Hamburg, Germany; German Center for Cardiovascular Research (DZHK) Partner Site Hamburg-Kiel-Lübeck, Hamburg, Germany; Department of Cardiology, University Heart and Vascular Center Hamburg-Eppendorf, University Medical Center Hamburg-Eppendorf, Hamburg, Germany; German Center for Cardiovascular Research (DZHK) Partner Site Hamburg-Kiel-Lübeck, Hamburg, Germany; III. Department of Medicine, University Medical Center Hamburg-Eppendorf, Hamburg, Germany; Hamburg Center for Kidney Health (HCKH), University Medical Center Hamburg-Eppendorf, Hamburg, Germany; Department of Internal Medicine, Section Gender- and Sex-Specific Medicine and Prevention, Otto-Von-Guericke University Magdeburg, Magdeburg, Germany; German Society of Gender-Specific Medicine, Potsdam, Germany; III. Department of Medicine, University Medical Center Hamburg-Eppendorf, Hamburg, Germany; Hamburg Center for Kidney Health (HCKH), University Medical Center Hamburg-Eppendorf, Hamburg, Germany; III. Department of Medicine, University Medical Center Hamburg-Eppendorf, Hamburg, Germany; Hamburg Center for Kidney Health (HCKH), University Medical Center Hamburg-Eppendorf, Hamburg, Germany

**Keywords:** awareness, blood pressure, hypertension, menopausal transition, sex differences

## Abstract

**Background:**

Sex differences in hypertension epidemiology and management are increasingly recognized, yet their variation across the adult life course remains incompletely understood. We examined sex-specific differences in hypertension prevalence, awareness, treatment, and blood pressure (BP) control in a German population.

**Methods:**

This cross-sectional analysis included 10 000 participants (51% women; mean age 62 ± 8.5 years) from the Hamburg City Health Study. Hypertension was defined per 2023 European Society of Hypertension guidelines. Awareness, treatment, and BP control were defined based on self-reported diagnosis, current medication use, and treated BP below both thresholds. Age-stratified analyses (45–54, 55–64, and 65–74 years) allowed direct sex comparisons. Differences were assessed using modified Poisson regression adjusted for sociodemographic, comorbidity, and lifestyle factors.

**Results:**

Hypertension prevalence was higher in men than women (71% vs. 57%), particularly at 45–54 years [50% vs. 32%; adjusted prevalence ratio (PR) 1.44, 95% confidence interval (CI) 1.27–1.62]. Sex differences progressively attenuated with age (PR 1.23, 95% CI 1.15–1.32 at 55–64 years) and converged at 65–74 years (PR 1.04, 95% CI 0.99–1.08). Findings were consistent in analyses based on self-reported menopausal status. Awareness and treatment were similar between sexes. BP control remained low (∼40%) and declined in women at 65–74 years.

**Conclusion:**

Hypertension prevalence was high and BP control suboptimal. Sex differences in hypertension prevalence varied across the adult life course, with higher prevalence among men at younger ages and convergence after age 55, coinciding with menopausal age. These findings support sex-specific, life stage–oriented hypertension screening and management strategies.

KEY LEARNING POINTS **What was known:**

- Sex differences in hypertension are frequently investigated without accounting for variations across the adult life course, leaving the evidence base for sex-sensitive guidelines insufficient.
- Sex differences in BP and hypertension prevalence are known to be dynamic, but the contributions of biological versus socioeconomic and lifestyle factors remain incompletely understood.
- Evidence on sex-specific hypertension awareness, treatment, and control remains limited in European populations and rarely accounts for life-stage transitions such as menopause.

**This study adds:**

- In a large, population-based German cohort, hypertension prevalence was high in both sexes (71% in men, 57% in women overall), with substantially lower BP control than reported in US cohorts.
- Sex differences in hypertension prevalence and BP were most pronounced in younger adults, formally confirmed by sex × age interaction models, and converged at ages 65–74 years, independent of comorbidities and lifestyle factors.
- BP control was low across all life stages and declined in women at older ages (65–74 years); men received slightly more antihypertensive medications and were more frequently prescribed ACE inhibitors and calcium channel blockers.

**Potential impact:**

- These findings support sex-specific, life stage–oriented hypertension screening and management, identifying the age window corresponding to the menopausal transition as a potentially critical period for intensified BP monitoring and prevention in women.
- Persistently high hypertension prevalence and low BP control across all life stages highlight the need for improved detection and treatment intensification strategies in European populations.

## INTRODUCTION

Despite growing awareness of sex differences in hypertension, data remain insufficient to develop truly sex-sensitive guidelines [1]. Women have historically been underrepresented in major interventional hypertension trials [2–4], and sex differences are frequently investigated without accounting for variations across the life course.

Given these limitations, observational studies provide an important opportunity to investigate sex differences in hypertension epidemiology. A recent analysis from the Atherosclerosis Risk in Communities Study provides contemporary data on sex differences in hypertension, treatment, and control in the US [5]. Prior evidence indicates that sex differences in blood pressure (BP) are dynamic across the life course [6], yet lifespan-specific differences between sexes remain incompletely understood. It also remains unclear to what extent sex differences reflect underlying biological factors versus differences in socioeconomic characteristics or key risk factors, including modifiable lifestyle behaviors.

The role of the menopausal transition in sex differences in hypertension is also not well-defined. Menopause is associated with substantial cardiometabolic changes that may influence BP [7] and hormone-replacement therapy (HRT) has been shown to affect these processes, although its impact on BP remains controversial [8]. Although most evidence on sex differences in hypertension epidemiology has historically derived from large US cohorts, hypertension prevalence has traditionally been higher in Europe and Asia, highlighting the need for data from these regions [5, 9–13]. While relevant European evidence is increasingly available, large-scale population-based data on sex differences in hypertension awareness, treatment, and control from Central Europe remain scarce [14, 15].

Therefore, this study aims to examine sex-specific differences in hypertension prevalence, awareness, treatment, and control in a large population-based German cohort, with particular focus on age-based stages across the adult life course. Secondary analyses additionally explore associations with menopausal status, modifiable lifestyle factors, and postmenopausal hormone replacement therapy (HRT) use.

## MATERIALS AND METHODS

### Study design and population

Data were derived from the Hamburg City Health Study (HCHS), an ongoing population-based cohort study described previously [16]. Briefly, the study comprises a randomly selected sample of residents from Hamburg, Germany, aged 45–74 years. All participants provided written informed consent, and the study followed the principles of the Declaration of Helsinki. The first 10 000 participants with complete data, enrolled between February 2016 and November 2018, were included in the present analysis.

### Data collection and definition of comorbidities

Participants completed a comprehensive study visit, including assessment of vital signs, laboratory analyses, and questionnaires [16, 17]. Data on demographics, smoking status (never, former, or current), menopausal status (assessed by standardized questionnaire in all female participants), physical activity (hours of sport per week), medication use, education, and diagnoses of coronary heart disease, myocardial infarction, chronic obstructive pulmonary disease (COPD), and malignancies were collected through standardized interviews. Education was categorized using the International Standard Classification of Education [18]. Dyslipidemia was defined by lipid-lowering medication use or a low-density to high-density lipoprotein ratio >3.5. Diabetes was defined by antidiabetic drug use, fasting blood glucose ≥126 mg/dl, non-fasting blood glucose ≥200 mg/dl, HbA1c ≥6.5%, or self-reported diagnosis. Heart failure was diagnosed according to the 2021 European Society of Cardiology (ESC) guidelines [19]. The estimated glomerular filtration rate (eGFR) was calculated using the 2009 Chronic Kidney Disease Epidemiology Collaboration formula based on creatinine (CKD-Epi_Creat_), with chronic kidney disease (CKD) defined as an eGFR <60 ml/min/1.73 m² [20]. Laboratory analyses were performed as described [21, 22]. Dietary sodium and potassium intake were estimated using the Kawasaki formula from a second-morning spot urine sample [23].

### Blood pressure measurements

BP was measured by trained study nurses following standardized procedures [17]. After 5 min of rest, two readings were taken at least 2 min apart using digital automatic monitors (boso-medicus uno, BOSCH + SOHN, Jungingen, Germany); systolic (SBP) and diastolic BP (DBP) were calculated as the mean. Participants were not informed of their readings. This protocol was consistent with ESH/ESC guideline recommendations applicable at the time of study design and recruitment.

### Definition of hypertension, awareness, and control

Hypertension was defined according to the 2023 European Society of Hypertension (ESH) guideline as SBP ≥140 mmHg, DBP ≥90 mmHg, or antihypertensive medication use [24]. Awareness was defined as self-reported knowledge of a hypertension diagnosis. Treatment was defined as current antihypertensive medication use irrespective of BP level. BP control was defined as treating BP below both thresholds (<140 mmHg and <90 mmHg for SBP, and DBP).

### Definition of life-stage and menopausal subgroups

Based on the cohort-specific distribution of menopausal status (Fig. S1), participants were classified into three age-based life-stage groups: 45–54, 55–64, and 65–74 years [25], allowing direct sex comparisons within identical age strata. A sensitivity analysis using self-reported menopausal status classified women as pre-/perimenopausal or postmenopausal [further subdivided into early (age <65) and late (age ≥65) postmenopause]. Women with missing menopausal status (*n* = 521; 10.2%) were excluded from this analysis. Men were weighted to the respective female groups using inverse probability weighting.

### Statistical analyses

Continuous variables are presented as mean ± standard deviation (SD) or median with interquartile range (IQR), as appropriate. Categorical variables are reported as counts and percentages. Sex differences in hypertension prevalence, awareness, treatment, and BP control were estimated using modified Poisson regression models with robust standard errors, yielding prevalence ratios (PRs) with 95% confidence intervals (CIs). Physical activity was log-transformed prior to analysis. Three adjustment models were applied: Model A included sociodemographic variables (age, education), Model B adjusted for comorbidities (additionally: diabetes, dyslipidemia, CKD), and Model C further incorporated modifiable lifestyle factors (additionally: smoking, physical activity, body mass index, sodium and potassium intake). Age was modeled using restricted cubic splines with 4 degrees of freedom. To formally assess whether age-related trajectories differed by sex, spline models with and without a sex × age interaction term were compared using likelihood ratio tests. Missing data (Table S1) were imputed using random forest-based imputation (missForest version 1.4). Analyses were conducted in R version 4.5.2.

## RESULTS

### Baseline characteristics

The mean age was 62 ± 8.5 years, and 51% were women (Table 1). Dyslipidemia (22.8%), malignancies (16%), CKD (11.6%), diabetes (8%), and COPD (6.3%) were the most prevalent comorbidities. Men more frequently had dyslipidemia, diabetes, CKD, coronary heart disease, and heart failure, whereas malignancies and COPD were more common among women. Baseline characteristics across age-based life-stage groups and menopausal status are shown in Tables S2 and S3.

**Table 1:** Baseline characteristics.

Characteristics	Overall (*n* = 10 000)	Women (*n* = 5108)	Men (*n* = 4892)
Demographics			
Age in years (mean ± SD)	62.3 ± 8.5	62 ± 8.4	62.8 ± 8.5
BMI in kg/m² (mean ± SD)	26.6 ± 4.6	26.2 ± 5.0	27.3 ± 4.1
Weight in kg (mean ± SD)	78.7 ± 15.9	71.2 ± 13.9	86.4 ± 14.0
Heart rate in bpm (mean ± SD)	69.7 ± 10.9	70.5 ± 10.2	68.9 ± 11.6
Ethnicity [*n* (%)]			
Caucasian	9802 (98)	5014 (98.2)	4788 (97.9)
Asian	57 (0.6)	31 (0.6)	26 (0.5)
Black	36 (0.4)	12 (0.2)	24 (0.5)
Hispanic	35 (0.4)	19 (0.4)	16 (0.3)
Other	40 (0.4)	19 (0.4)	21 (0.4)
Unknown	30 (0.3)	13 (0.2)	17 (0.3)
Comorbidities [*n* (%)]			
Dyslipidemia	2277 (22.8)	855 (16.7)	1427 (29.1)
Malignancies	1600 (16.0)	869 (17.0)	731 (14.9)
Diabetes	801 (8.0)	306 (6.0)	495 (10.1)
COPD	629 (6.3)	370 (7.2)	259 (5.3)
Chronic kidney disease	1169 (11.6)	497 (9.7)	672 (13.7)
Coronary heart disease	517 (5.2)	117 (2.3)	400 (8.2)
Heart failure	337 (3.4)	126 (2.5)	211 (4.3)
Socioeconomics [*n* (%)]			
Employment			
Full-time	3566 (35.7)	1337 (26.2)	2229 (45.6)
Part-time	1409 (14.1)	1095 (21.4)	314 (6.4)
Unemployed or retired	5025 (50.3)	2676 (52.4)	2349 (48.0)
Education			
High	4429 (44.3)	1763 (34.5)	2666 (54.5)
Medium	5105 (51.1)	3037 (59.5)	2068 (42.3)
Low	466 (4.7)	308 (6.0)	158 (3.2)
Lifestyle			
Smoking status [*n*, (%)]			
Current	1984 (19.8)	1001 (19.6)	983 (20.1)
Former	4434 (44.3)	2040 (39.9)	2394 (48.9)
Never	3582 (35.8)	2067 (40.5)	1515 (31.0)
Physical activity (sports in hours per week) [median (IQR)]	2 (0, 3.5)	2 (1, 3.5)	2 (0, 3.6)
Sodium intake in g/d (mean ± SD)	4.5 ± 1.4	4.2 ± 1.2	4.9 ± 1.4
Salt (sodium chloride) intake in g/d (mean ± SD)	11.4 ± 3.5	10.6 ± 3.0	12.4 ± 3.5
Potassium intake in g/d (mean ± SD)	2.7 ± 0.5	2.6 ± 0.5	2.9 ± 0.6
Medication [*n* (%)]			
Lipid-lowering medication	1733 (17.3)	678 (13.3)	1055 (21.6)
Laboratory results			
Hemoglobin in g/dl (mean ± SD)	14.3 ± 1.1	13.7 ± 0.9	15.0 ± 1.0
Sodium in mmol/l (mean ± SD)	139 ± 2	139 ± 2	139 ± 2
Potassium in mmol/l (mean ± SD)	3.9 ± 0.3	3.8 ± 0.3	3.9 ± 0.3
Creatinine in mg/dl (mean ± SD)	0.87 ± 0.22	0.77 ± 0.14	0.97 ± 0.24
hsCRP in mg/dl [median (IQR)]	0.12 (0.06, 0.27)	0.12 (0.06, 0.27)	0.12 (0.06, 0.26)
TSH in mU/ml [median (IQR)]	1.19 (0.83, 1.64)	1.17 (0.80, 1.63)	1.20 (0.85, 1.65)

BMI, body mass index; COPD, chronic obstructive pulmonary disease; hsCRP, high-sensitive C-reactive protein; IQR, interquartile range; TSH, thyroid-stimulating hormone; SD, standard deviation.

### Hypertension and BP trajectories by sex

**Figure 1:**
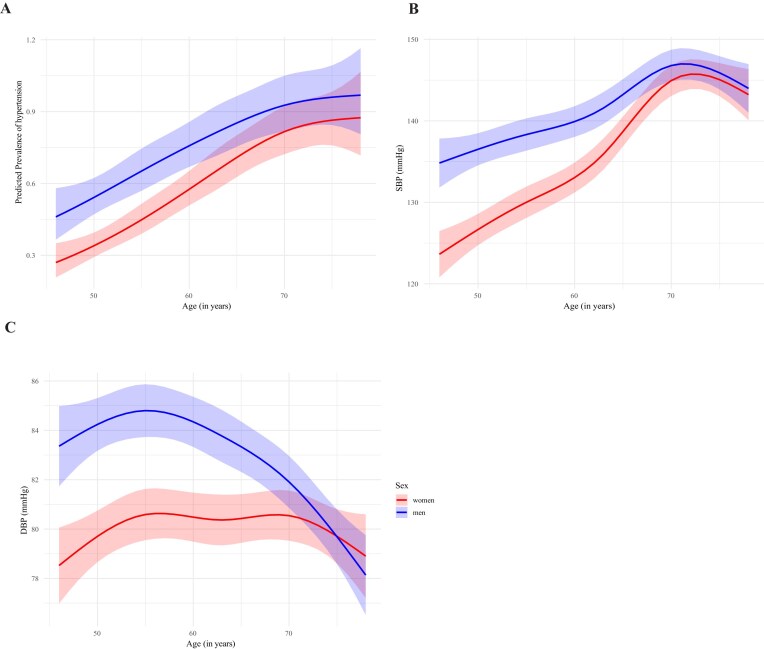
Sex-specific age trajectories of hypertension, SBP, and DBP. Panel A shows age-specific prevalence ratios (PRs) for hypertension estimated using Poisson regression models with robust variance. Hypertension prevalence, defined as SBP ≥140 mmHg, DBP ≥90 mmHg, or use of antihypertensive medication, increases with age in both sexes, with men having a higher prevalence than women at younger ages; the difference narrows with increasing age. Panel B shows mean SBP levels by sex, following a similar trajectory to hypertension prevalence. Panel C shows mean DBP levels by sex, with higher levels in younger men that decline with advancing age, approaching levels in women. Shaded areas indicate 95% confidence intervals (CIs). Models were adjusted for age and education.

Overall hypertension prevalence was 71% in men and 57% in women. Prevalence increased steadily with age in both sexes, but trajectories differed across the age spectrum (Fig. 1A). Formal comparison of spline models with and without sex × age interaction terms demonstrated significant sex differences in age-related trajectories for hypertension prevalence, SBP, and DBP (all *P* < .001; Table S4). At younger and middle age, hypertension prevalence was higher in men. With advancing age, this difference visually narrowed, and prevalence in women appeared to approach that in men by ∼70 years.

Mean SBP was 142 ± 18.2 mmHg in men and 136 ± 19.5 mmHg in women, showing similar age-related patterns as observed for hypertension (Fig. 1B). Sex differences were greatest at younger and middle age. From ∼60 years onward, the prevalence of higher SBP values appeared to increase more steeply in women, progressively approaching levels observed in men.

Mean DBP was 84 ±10 mmHg in men and 81 ± 9.6 mmHg in women (Fig. 1C). In both sexes, the prevalence of higher DBP values visually increased until ∼55 years. Thereafter, DBP prevalence appeared to remain stable in women but decline steadily in men.

### Sex differences in BP and hypertension across life-stages

Among younger adults (45–54 years), hypertension prevalence was significantly higher in men (50% vs. 32% in women; Table 2). This difference remained after adjustment for socioeconomics, comorbidities, and modifiable lifestyle factors (fully adjusted PR 1.44, 95% CI 1.27–1.62). Adjusted SBP and DBP levels were 8.1 mmHg (95% CI 6.6–9.5) and 4.0 mmHg (95% CI 3.2–4.9) higher in men than in women within this life-stage, respectively (Table 3).

**Table 2:** Sex-specific hypertension prevalence across life-stages.

	Prevalence men(95% CI)	Prevalence women(95% CI)	PR (95% CI)Model A	PR(95% CI)Model B	PR(95% CI) Model C
All(*n* = 10 000)	0.71(0.69, 0.72)	0.57(0.56, 0.58)	1.25(1.22, 1.29)	1.20(1.17, 1.24)	1.16(1.12, 1.20)
45–54 years(*n* = 2273)	0.50(0.47, 0.53)	0.32(0.30, 0.34)	1.56(1.41, 1.72)	1.49(1.35, 1.65)	1.44(1.27, 1.62)
55–64 years(*n* = 3322)	0.69(0.67, 0.71)	0.51(0.49, 0.53)	1.35(1.28, 1.43)	1.29(1.22, 1.36)	1.23(1.15, 1.32)
65–74 years(*n* = 4405)	0.84(0.82, 0.85)	0.75(0.72, 0.76)	1.12(1.09, 1.16)	1.08(1.04, 1.11)	1.04(0.99, 1.08)

Prevalence is outlined by sex across life-stages. Sex-differences, given as prevalence ratios (PRs) with 95% confidence intervals (CIs) between men and women, were analyzed by Poisson regression models with robust variance estimation. Model A was adjusted for age and education; Model B for age, education, diabetes, chronic kidney disease, and dyslipidemia; and Model C for age, education, diabetes, chronic kidney disease, dyslipidemia, smoking status, physical activity, body mass index, sodium intake, and potassium intake. CI, confidence interval; PR, prevalence ratio.

**Table 3:** Sex-specific blood pressure profiles across life-stages.

	Mean men(95% CI)	Mean women(95% CI)	Mean difference (95%-CI)Model A	Mean difference (95%-CI)Model B	Mean difference(95%-CI)Model C
SBP					
All(*n* = 10 000)	142(141, 142)	136(136, 137)	5.5(4.7, 6.2)	5.2(4.5, 6.0)	4.4(3.5, 5.2)
45–54 years(*n* = 2273)	137(136, 137)	127(126, 128)	9.6(8.3, 10.8)	9.2(8.0, 10.5)	8.1(6.6, 9.5)
55–64 years(*n* = 3322)	140(139, 141)	133(132, 134)	7.1(5.9, 8.3)	6.7(5.5, 7.9)	5.5(4.1, 6.9)
65–74 years(*n* = 4405)	146(145, 147)	144(143, 145)	2.1(0.9, 3.3)	2.0(0.8, 3.2)	1.5(0.6, 2.8)
DBP					
All(*n* = 10 000)	84(83, 84)	81(80, 81)	2.9(2.5, 3.3)	3.0(2.6, 3.4)	3.0(2.5, 3.4)
45–54 years(*n* = 2273)	85(84, 85)	80(80, 81)	4.5(3.7, 5.3)	4.4(3.6, 5.2)	4.0(3.2, 4.9)
55–64 years(*n* = 3322)	85(84, 85)	81(81, 82)	3.8(3.1, 4.5)	3.7(3.0, 4.4)	3.6(2.8, 4.3)
65–74 years(*n* = 4405)	82(82, 83)	81(81, 81)	1.2(0.6, 1.8)	1.6(0.9, 2.2)	1.9(1.1, 2.6)

Mean systolic and diastolic blood pressure levels are shown by sex across life-stages. Sex-differences were analyzed using linear regression models. Model A was adjusted for age and education; Model B for age, education, diabetes, chronic kidney disease, and dyslipidemia; and Model C for age, education, diabetes, chronic kidney disease, dyslipidemia, smoking status, physical activity, body mass index, sodium intake, and potassium intake. DBP, diastolic blood pressure; SBP, systolic blood pressure; CI, confidence interval.

During middle age (55–64 years), hypertension prevalence remained significantly higher in men than in women, though the magnitude of this difference attenuated (fully adjusted PR 1.23, 95% CI 1.15–1.32; Table 2), Sex differences in BP levels likewise remained significant but differences attenuated, with an adjusted difference of 5.5 mmHg, (95% CI 4.1–6.9) for SBP and 3.6 mmHg (95% CI 2.8–4.3) for DBP (Table 3).

At older age (65–74 years), hypertension prevalence no longer differed significantly between sexes (84% in men and 75% in women; fully adjusted PR 1.04, 95% CI 0.99–1.08) and BP levels converged with a mean adjusted difference of 1.5 mmHg (95% CI 0.6–2.8) for SBP and 1.9 mmHg (95% CI 1.1–2.6) for DBP (Table 3).

Higher BMI, sodium intake, and lower physical activity were similarly associated with higher hypertension prevalence in both sexes, with associations strongest at younger ages (Fig. S2). Sensitivity analyses using self-reported menopausal status yielded results comparable to the primary age-stratified analyses (Table S5). Hypertension prevalence and BP levels did not differ between postmenopausal women with and without HRT (Table S6).

### Sex differences in awareness, treatment, and control

**Figure 2:**
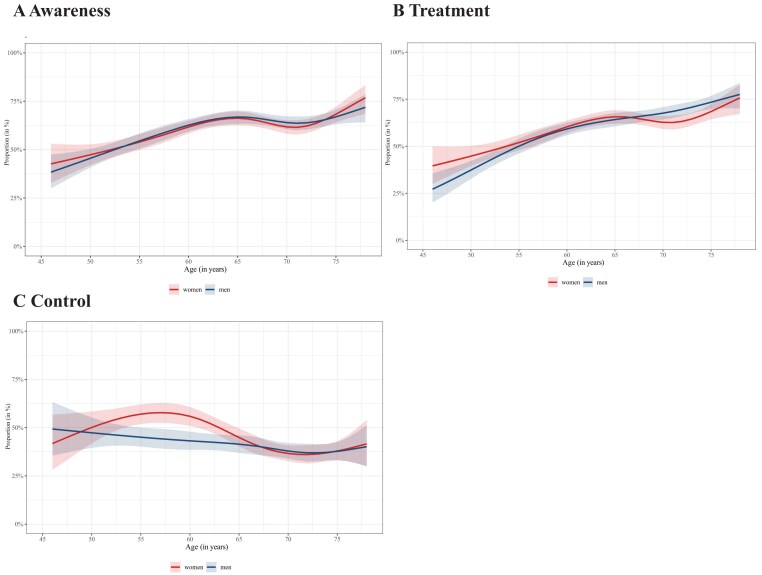
Sex-specific age trajectories of awareness, treatment, and control. Panel A shows prevalence ratios (PRs) for hypertension awareness and Panl B for hypertension treatment, both among all individuals with hypertension. Awareness and treatment similarly increases with age in both sexes. Panel C shows PRs for hypertension control among hypertensive individuals receiving blood pressure lowering treatment. Hypertension control was low in both sexes. PRs were estimated using Poisson regression models with robust variance. Shaded areas indicate 95% confidence intervals (CIs). Models were adjusted for age and education.

Awareness and treatment increased with age with comparable prevalences and trajectories between sexes (Fig. 2A, B). In younger adults (45–54 years), 46% of hypertensive women were aware of their condition and 44% received treatment, compared with 42% and 36% in men. At older ages (65–74 years), awareness and treatment prevalence increased to 63% and 64% in women and to 62% and 66% in men, respectively (Table S7).

In contrast, patterns differed for BP control (Fig. 2C). In men, the proportion achieving BP control remained ∼40% across life stages. In women, BP control prevalence increased from younger to middle age, reaching 56%—exceeding men at this stage (age- and education-adjusted PR 0.78, 95% CI 0.69–0.88)—but declined to 38% at older ages, comparable to men (adjusted PR 1.03, 95% CI 0.92–1.15; Table S7). Men with hypertension received slightly more antihypertensive medications (adjusted PR 1.10, 95% CI 1.04–1.16) and were more frequently prescribed ACE inhibitors (PR 1.33, 95% CI 1.21–1.46) and calcium antagonists (PR 1.27, 95% CI 1.12–1.44; Table S8).

## DISCUSSION

In this large population-based cohort, hypertension prevalence was high in both sexes, while the proportion of treated individuals achieving BP control was low. Compared with US population-based data, hypertension prevalence and SBP appeared higher in our cohort: the National Health and Nutrition Examination Survey (NHANES) reported a hypertension prevalence of ∼50%, using a three-reading BP protocol, compared with 64% in our study applying two BP readings [26], and ∼70% of treated individuals achieved BP control in contemporary US data of the NHANES compared with only 42% in our cohort [27]. Although these differences are partly attributable to measurement protocols and population characteristics, the observed differences remain substantial and consistent with previously reported European–North American differences, potentially reflecting variations in lifestyle factors, population characteristics, or healthcare systems [9, 28]. Against this background of high overall burden, we observed significant sex differences in hypertension prevalence and BP profiles that varied across the adult life course. At younger ages, BP levels and hypertension prevalence were higher in men than in women. These differences progressively attenuated with advancing age, as evidenced by declining prevalence ratios for hypertension across life stages and narrowing of mean BP differences between sexes. These sex-specific differences in BP levels were accompanied by differences in BP control, with higher BP control in women at younger ages that converged with those observed in men at older ages.

Men showed markedly higher hypertension prevalence and SBP at younger ages (45–54 years), with an approximately 25% higher hypertension prevalence and 8 mmHg higher SBP compared with women. Although these differences diminished with age, they remained evident until ∼70 years. Importantly, this sex-difference persisted after adjustment for sociodemographic characteristics, comorbidities, and key lifestyle factors in our study, pointing toward a contribution of sex-specific biological factors—consistent with findings from the French CONSTANCES cohort and the UK Biobank, showing higher hypertension prevalence in men than women [14, 15]. These findings indicate that screening strategies targeting men at midlife may be particularly beneficial, as awareness and treatment were lowest in this age group. In addition, lifestyle factors such as BMI, sodium intake, and physical activity showed the strongest associations with hypertension at younger ages, highlighting the potential impact of lifestyle interventions during this life stage.

In contrast, women showed a more pronounced increase in mean SBP values and hypertension prevalence across age, resulting in convergence at older ages—with the largest attenuation of sex differences occurring between middle (55–64) and older age (65–74), independent of other risk factors. Importantly, the applied age-based life stages closely approximated the menopausal transition in the participating women, and sensitivity analyses stratifying by self-reported menopausal status yielded results comparable to the primary age-stratified analyses, suggesting a contribution of biological processes beyond chronological aging. The loss of estrogen during menopause has been linked to several BP–regulating pathways [29–33]. In addition, increased sympathetic activation after menopause has been reported and may further contribute to rising BP levels [34, 35]. Beyond these changes, menopause is also associated with adverse changes in body fat distribution, insulin sensitivity, and vascular function, which may further contribute to increasing BP levels [36]. Notably, BP did not differ between postmenopausal women with and without HRT. While this observation should be interpreted with caution due to potential confounding by indication and lack of detailed information on timing and duration of therapy, it might suggest contributions from factors beyond estrogen decline.

DBP declined progressively in men after ∼55 years, whereas it remained relatively stable in women. This diverging trajectory is consistent with sex differences in vascular aging: the earlier and steeper rise in large-artery stiffness observed in men may underlie their accelerated DBP decline after midlife [37], while women’s vascular aging accelerates after menopause, manifesting primarily as rising SBP [38]. This pattern is clinically relevant, as a widening pulse pressure—the consequence of rising SBP and stable or declining DBP—is an independent predictor of cardiovascular risk, particularly in older women [39, 40].

Despite increasing awareness and treatment with age, the proportion of treated individuals achieving BP control was also alarmingly low. Only 42% of individuals receiving antihypertensive treatment achieved BP control, and the BP control advantage observed in women at younger ages attenuated progressively with age. These findings are not readily explained by differences in antihypertensive prescribing, as the number of antihypertensive medications was even slightly higher in treated men compared with women. This discrepancy may reflect insufficient treatment intensification, therapeutic inertia, adherence differences, or delayed diagnosis and treatment initiation.

Key strengths of this study include its large population-based sample, balanced sex distribution across a broad range of life stages, comprehensive phenotyping encompassing lifestyle factors and menopausal status, as well as a highly standardized BP measurement. Still, several limitations should be considered. First, the cross-sectional design precludes conclusions about causality or temporal relationships and the observed age-related changes in BP may reflect cohort effects rather than within-individual trajectories. Due to the cross-sectional design, survivor bias due to premature cardiovascular mortality in men cannot be excluded and may have contributed to the observed convergence of BP levels at older ages. Second, the analysis of HRT was observational and subject to potential confounding by indication. Third, only two BP readings were obtained. Although this approach followed guideline recommendations at the time of study initiation, current guidelines recommend an additional reading [41, 42]. Furthermore, BP was measured in the presence of study nurses and out-of-office (ambulatory or home) validation was not available. Thus, white-coat effects, including potential sex-specific differences, cannot be excluded. Also, hypertensive disorders of pregnancy, which are established risk factors for later-life hypertension and cardiovascular disease [43], were not incorporated into the primary analyses because reproductive health data were available only in a very small subset of female participants.

The lack of 24-h urine collections for more precise estimation of salt intake constitutes another limitation. Finally, although the population was randomly recruited from the general population with comparable characteristics to other population-based German studies, the cohort was primarily urban and ethnically homogeneous, limiting generalizability to more diverse or rural populations.

In summary, this large population-based cohort revealed a high burden of hypertension with specific sex differences that varied across the adult life course. While men exhibited higher BP levels during midlife, women experienced a marked increase in hypertension across age ranges corresponding to the menopausal transition, with convergence of BP profiles at older ages. Despite increasing awareness and treatment, hypertension control remained suboptimal across all life stages. These findings emphasize that sex differences in hypertension are dynamic rather than static and highlight the need for sex-specific and life stage–oriented approaches to screening and prevention.

## Supplementary Material

sfag180_Supplemental_File

## Data Availability

Data will be shared upon reasonable request to the corresponding author. Data-sharing is subject to the approval of the steering committee of the Hamburg City Health Study.
