# Supplementary material for: Sex differences in blood pressure and hypertension through adulthood
Source: Clin Kidney J. 2026 Jun 4;19(8):sfag180. doi: 10.1093/ckj/sfag180 (PMC13424595; doi:10.1093/ckj/sfag180)
Supplement: sfag180_Supplemental_File [file sfag180_supplemental_file.docx]

**Supplementary Material**

| Table of contents | Page |
| --- | --- |
| Table S1. Number of missing values. | 2 |
| Figure S1. Age distribution of women according to self-reported menopausal status. | 3 |
| Table S2. Baseline characteristics stratified by age-based life-stages. | 4-5 |
| Table S3. Baseline characteristics for women by self-reported menopausal status. | 6-7 |
| Table S4. Sex differences in age-related trajectories of blood pressure and hypertension prevalence. | 8 |
| Figure S2. Sex-specific association of modifiable lifestyle factors with hypertension by age. | 9-10 |
| Table S5. Sensitivity analysis of hypertension prevalence and blood pressure by self-reported menopausal status in women and age-matched men. | 11-12 |
| Table S6. Blood pressure levels and hypertension prevalence in postmenopausal women stratified by hormone replacement therapy. | 13-14 |
| Table S7. Sex differences in awareness, treatment, and control across age-based life-stages. | 15 |
| Table S8. Sex differences in antihypertensive drug treatment. | 16 |

**Supplementary Table S1. Number of missing values.**

| **Characteristics, n (%)** | **Missing values** |
| --- | --- |
| Age | 0 (0) |
| Sex | 0 (0) |
| BMI | 560 (5.6) |
| Heart rate | 410 (4.1) |
| Systolic blood pressure | 410 (4.1) |
| Diastolic blood pressure | 410 (4.1) |
| Dyslipidemia | 710 (7.1) |
| Malignancies | 760 (7.6) |
| Diabetes | 790 (7.9) |
| COPD | 930 (9.3) |
| Chronic kidney disease | 750 (7.5) |
| Coronary heart disease | 150 (1.5) |
| Heart failure | 322 (4.1) |
| Employment | 1170 (11.7) |
| Education | 580 (5.8) |
| Smoking status | 50 (0.5) |
| Sport | 1450 (14.5) |
| Medication | 470 (4.7) |
| Hemoglobin | 310 (3.1) |
| Sodium | 300 (3) |
| Potassium | 300 (3) |
| Creatinine | 320 (3.2) |
| hsCRP | 380 (3.8) |
| TSH | 380 (3.8) |
| Urinary sodium-creatinine-ratio | 1600 (16) |
| Urinary potassium-creatinine-ratio | 1600 (16) |
| Menopausal status among women | 521 (10.2) |

BMI: body mass index; COPD: chronic obstructive pulmonary disease; hsCRP: high-sensitive C-reactive protein; TSH: Thyroid-stimulating hormone.

**Figure S1. Age distribution of women according to self-reported menopausal status.**

Age distribution among women stratified by self-reported menopausal status (pre-, peri-, and postmenopausal).

**Supplementary Table S2. Baseline characteristics stratified by age-based life-stages.**

|  | **Women** | | | **Men** | | |
| --- | --- | --- | --- | --- | --- | --- |
|  | **45–54 years (n = 1227)** | **55–64 years**  **(n = 1745)** | **65–74 years**  **(n = 2136)** | **45–54 years (n = 1046)** | **55–64 years**  **(n = 1577)** | **65–74 years**  **(n = 2269)** |
| **Demographics** | | | |  |  |  |
| Age in years (mean ± SD) | 50.7 ± 2.3 | 59.5 ± 2.9 | 70.4 ± 3.5 | 50.7 ± 2.3 | 59.7 ± 2.9 | 70.6 ± 3.6 |
| BMI in kg/m² (mean ± SD) | 25.6 ± 5.0 | 26.4 ± 5.1 | 26.6 ± 4.8 | 27.0 ± 4.2 | 27.3 ± 4.3 | 27.4 ± 4.0 |
| **Comorbidities (n (%))** | | | | | | |
| Dyslipidemia | 84 (6.8) | 242 (13.9) | 529 (24.8) | 168 (16.1) | 406 (25.7) | 850 (37.5) |
| Malignancies | 111 (9.0) | 239 (13.7) | 519 (24.3) | 54 (5.2) | 149 (9.4) | 525 (23.1) |
| Diabetes | 33 (2.7) | 97 (5.6) | 179 (8.4) | 52 (5.0) | 118 (7.5) | 327 (14.4) |
| COPD | 67 (5.5) | 119 (6.8) | 184 (8.6) | 38 (3.6) | 88 (5.6) | 133 (5.9) |
| Chronic kidney disease | 10 (0.8) | 45 (2.6) | 214 (10.0) | 8 (0.8) | 51 (3.2) | 280 (12.3) |
| Coronary heart disease | 3 (0.2) | 11 (0.6) | 42 (2.0) | 17 (1.6) | 50 (3.2) | 187 (8.2) |
| Heart failure | 7 (0.6) | 23 (1.3) | 94 (4.4) | 13 (1.2) | 32 (2.0) | 167 (7.4) |
| **Socioeconomics (n (%))** | | | | | | |
| Employment |  |  |  |  |  |  |
| Full-time | 608 (49.6) | 691 (39.6) | 52 (2.4) | 943 (90.2) | 1125 (71.3) | 158 (7) |
| Part-time | 501 (40.8) | 625 (35.8) | 197 (9.2) | 58 (5.5) | 153 (9.7) | 215 (9.5) |
| Unemployed or retired | 118 (9.6) | 429 (24.6) | 1887 (88.3) | 45 (4.3) | 299 (19) | 1896 (83.6) |
| Education |  |  |  |  |  |  |
| High | 550 (44.8) | 699 (40.1) | 525 (24.6) | 560 (53.5) | 876 (55.5) | 1239 (54.6) |
| Medium | 641 (52.2) | 960 (55.0) | 1425 (66.7) | 450 (43.0) | 648 (41.1) | 961 (42.4) |
| Low | 36 (2.9) | 86 (4.9) | 186 (8.7) | 36 (3.4) | 53 (3.4) | 69 (3.0) |
| **Lifestyle** | | | | | | |
| Smoking status (n, (%)) |  |  |  |  |  |  |
| Current | 323 (26.3) | 398 (22.8) | 280 (13.1) | 284 (27.2) | 386 (24.5) | 313 (13.8) |
| Former | 399 (32.5) | 725 (41.5) | 918 (43.0) | 386 (36.9) | 699 (44.3) | 1306 (57.6) |
| Never | 505 (41.2) | 622 (35.6) | 938 (43.9) | 376 (35.9) | 492 (31.2) | 650 (28.6) |
| Physical activity (sports in hours per week (median (IQR)) | 2 (1, 3.6) | 2 (0.5, 3.5) | 2.3 (0.3, 4) | 2 (0, 4) | 2 (0, 3.5) | 2.1 (0, 4) |
| Sodium intake in g/d (mean ± SD) | 4.2 ± 1.1 | 4.1 ± 1.3 | 4.0 ± 1.3 | 5.1 ± 1.6 | 4.9 ± 1.4 | 4.7 ± 1.3 |
| Salt (sodium chloride) intake in g/d (mean ± SD) | 10.5 ± 2.8 | 10.4 ± 3.3 | 10.3 ± 3.3 | 12.8 ± 4 | 12.3 ± 3.5 | 11.8 ± 3.3 |
| Potassium intake in g/d (mean ± SD) | 2.6 ± 0.5 | 2.6 ± 0.5 | 2.5 ± 0.5 | 3.1 ± 0.7 | 2.9 ± 0.6 | 2.8 ± 0.5 |
| **Laboratory Results** | | | | | | |
| Hemoglobin in g/dL (mean ± SD) | 13.6 ± 0.9 | 13.8 ± 0.8 | 13.8 ± 0.9 | 15.2 ± 0.9 | 15.1 ± 0.9 | 14.8 ± 1.1 |
| Sodium in mmol/L (mean ± SD) | 139 ± 2 | 139 ± 2 | 139 ± 2.4 | 139 ± 2 | 139 ± 2.2 | 139 ± 2.4 |
| Potassium in mmol/L (mean ± SD) | 3.8 ± 0.3 | 3.8 ± 0.3 | 3.9 ± 0.3 | 3.9 ± 0.3 | 3.9 ± 0.3 | 3.9 ± 0.3 |
| Creatinine in mg/dL (mean ± SD) | 0.75 ± 0.11 | 0.76 ± 0.13 | 0.78 ± 0.17 | 0.93 ± 0.14 | 0.95 ± 0.16 | 1.01 ± 0.32 |
| hsCRP in mg/dL (median (IQR)) | 0.09 (0.04, 0.22) | 0.12 (0.06, 0.27) | 0.14 (0.08, 0.31) | 0.10 (0.05, 0.24) | 0.11 (0.06, 0.26) | 0.13 (0.07, 0.27) |
| TSH in mU/mL (median (IQR)) | 1.30 (0.90, 1.77) | 1.16 (0.82, 1.63) | 1.12 (0.73, 1.55) | 1.27 (0.90, 1.69) | 1.21 (0.85, 1.63) | 1.16 (0.83, 1.64) |

Baseline characteristics by age-based life-stages in women and men. BMI: body mass index; COPD: chronic obstructive pulmonary disease; hsCRP: high-sensitive C-reactive protein; IQR: interquartile range; SD: standard deviation; TSH: Thyroid-stimulating hormone.

**Supplementary Table S3. Baseline characteristics for women by self-reported menopausal status.**

| **Characteristics** | **Pre-/perimenopause**  **(n = 714)** | **Early menopause (n = 2047)** | **Late menopause**  **(n = 1826)** |
| --- | --- | --- | --- |
| **Demographics** | | | |
| Age in years (mean ± SD) | 50.7 ± 3.4 | 57.80 ± 4.2 | 70.3 ± 3.5 |
| BMI in kg/m² (mean ± SD) | 25.4 ± 4.8 | 26.2 ± 5.2 | 26.5 ± 4.7 |
| **Comorbidities (n (%))** | | | |
| Dyslipidemia | 36 (5.0) | 272 (13.3) | 451 (24.7) |
| Malignancies | 45 (6.3) | 296 (14.5) | 476 (26.1) |
| Diabetes | 13 (1.8) | 106 (5.2) | 150 (8.2) |
| COPD | 33 (4.6) | 142 (6.9) | 160 (8.8) |
| Chronic kidney disease | 8 (1.1) | 44 (2.1) | 180 (9.9) |
| Coronary heart disease | 2 (0.3) | 11 (0.5) | 33 (1.8) |
| Heart failure | 6 (0.8) | 23 (1.1) | 81 (4.4) |
| **Socioeconomics (n (%))** | | | |
| Employment |  |  |  |
| Full-time | 357 (50.0) | 834 (40.7) | 46 (2.5) |
| Part-time | 293 (41.1) | 763 (37.2) | 182 (9.9) |
| Unemployed or retired | 64 (9.0) | 450 (21.9) | 1598 (87.5) |
| Education |  |  |  |
| High | 360 (50.4) | 822 (40.2) | 489 (26.8) |
| Medium | 337 (47.2) | 1131 (55.3) | 1177 (64.5) |
| Low | 17 (2.4) | 94 (4.6) | 160 (8.8) |
| **Lifestyle** | | | |
| Smoking status (n, (%)) |  |  |  |
| Current | 155 (21.7) | 506 (24.7) | 242 (13.3) |
| Former | 242 (33.9) | 809 (39.5) | 785 (43.0) |
| Never | 2065 (40.4) | 317 (44.4) | 732 (35.8) |
| Physical activity (sports in hours per week (median (IQR)) | 2.1 (1.0, 3.8) | 2.0 (1.0, 3.5) | 2.2 (1.0, 4.0) |
| Sodium intake in g/d (mean ± SD) | 4.1 ± 1.1 | 4.1 ± 1.2 | 4.0 ± 1.3 |
| Salt (sodium chloride) intake in g/d (mean ± SD) | 10.3 ± 2.8 | 10.3 ± 3.0 | 10.0 ± 3.3 |
| Potassium intake in g/d (mean ± SD) | 2.6 ± 0.5 | 2.6 ± 0.5 | 2.5 ± 0.5 |
| **Laboratory Results** | | |  |
| Hemoglobin in g/dL (mean ± SD) | 13.6 ± 0.9 | 13.8 ± 0.8 | 13.8 ± 0.9 |
| Sodium in mmol/L (mean ± SD) | 139 ± 2.1 | 140 ±2.0 | 139 ± 2.4 |
| Potassium in mmol/L (mean ± SD) | 3.8 ± 0.2 | 3.8 ± 0.3 | 3.9 ± 0.3 |
| Creatinine in mg/dL (mean ± SD) | 0.75 ± 0.11 | 0.75 ± 0.13 | 0.78 ± 0.16 |
| hsCRP in mg/dL (median (IQR)) | 0.09 (0.04, 0.20) | 0.12 (0.06, 0.27) | 0.14 (0.08, 0.30) |
| TSH in mU/mL (median (IQR)) | 1.29 (0.90, 1.74) | 1.19 (0.84, 1.67) | 1.11 (0.72, 1.54) |

Baseline characteristics in women stratified by self-reported menopausal status. The postmenopausal status was further divided in early menopause (< 65 years), and late menopause (≥ 65 years). BMI: body mass index; COPD: chronic obstructive pulmonary disease; hsCRP: high-sensitive C-reactive protein; IQR: interquartile range; SD: standard deviation; TSH: Thyroid-stimulating hormone.

**Table S4. Sex differences in age-related trajectories of blood pressure and hypertension prevalence.**

| **Outcome** | **Test** | **df** | **Statistic** | **p-value** |
| --- | --- | --- | --- | --- |
| SBP | LRT | 4 | 21,171 | <0.001 |
| DBP | LRT | 4 | 4,389 | <0.001 |
| Hypertension prevalence | LRT | 4 | 22.77 | <0.001 |

Likelihood ratio tests were used to compare models with and without sex × age interaction terms for systolic blood pressure (SBP), diastolic blood pressure (DBP), and hypertension prevalence. For SBP and DBP, likelihood ratio statistics from linear regression models are reported; for hypertension prevalence, deviance statistics from Poisson regression models are shown. Owing to differences in model type and outcome scale, test statistics are not directly comparable (χ² for the continuous outcomes SBP/DBP, deviance statistics for hypertension prevalence). In all analyses, age was modelled using natural cubic splines with four degrees of freedom. All models were adjusted for education, dyslipidemia, diabetes, chronic kidney disease, BMI, physical activity, smoking, and sodium and potassium intake. Degrees of freedom (df), test statistics, and corresponding p-values are shown. Df: degrees of freedom; BP: blood pressure; DBP: diastolic blood pressure; SBP: systolic blood pressure; LRT: likelihood ratio test.

**Figure S2. Sex-specific association of modifiable lifestyle factors with hypertension by age.**

Associations were estimated using Poisson regression models with robust variance stratified by sex. Lines indicate estimates and shaded areas show 95% confidence intervals (CIs). Models were adjusted for age, education, diabetes, dyslipidemia, chronic kidney disease and the modifiable risk factors smoking, physical activity, BMI, sodium intake and potassium intake. BMI: body mass index.

**Table S5. Sensitivity analysis of hypertension prevalence and blood pressure by self-reported menopausal status in women and age-matched men.**

|  | Prevalence men  (95% CI) | Prevalence women  (95% CI) | PR  (95% CI) Model A | PR  (95% CI)  Model B | PR  (95% CI) Model C |
| --- | --- | --- | --- | --- | --- |
| Hypertension prevalence | | | | | |
| All  (n = 9479) | 0.69  (0.68, 0.71) | 0.56  (0.55, 0.58) | 1.26  (1.22, 1.30) | 1.21  (1.17, 1.25) | 1.16  (1.12, 1.20) |
| Pre-/peri-menopause  (n = 4743) | 0.50  (0.47, 0.53) | 0.33  (0.30, 0.37) | 1.51  (1.34, 1.70) | 1.46  (1.29, 1.64) | 1.35  (1.17, 1.55) |
| Early menopause  (n = 4670) | 0.64  (0.62, 0.66) | 0.47  (0.45, 0.50) | 1.38  (1.31, 1.45) | 1.32  (1.25, 1.40) | 1.26  (1.18, 1.35) |
| Late menopause  (n = 4095) | 0.82  (0.81, 0.84) | 0.76  (0.74, 0.78) | 1.12  (1.08, 1.15) | 1.08  (1.04, 1.11) | 1.03  (0.99, 1.08) |
|  | **Mean men**  **(95% CI)** | **Mean women**  **(95% CI)** | **Mean difference (95%-CI)**  **Model A** | **Mean difference (95%-CI)**  **Model B** | **Mean difference**  **(95%-CI)**  **Model C** |
| SBP | | | | | |
| All  (n = 9479) | 141  (141, 142) | 136  (136, 137) | 5.7  (5.0, 6.4) | 5.5  (4.7, 6.2) | 4.6  (3.8, 5.5) |
| Pre-/peri-menopause  (n = 4743) | 136  (136, 137) | 127  (126, 128) | 9.5  (8.2, 10.8) | 9.1  (7.8, 10.4) | 7.6  (6.2, 9.1) |
| Early menopause  (n = 4670) | 139  (139, 140) | 132  (131, 133) | 7.5  (6.4, 8.6) | 7.1  (6.0, 8.2) | 6.0  (4.8, 7.3) |
| Late menopause  (n = 4095) | 146  (145, 146) | 144  (143, 145) | 2.1  (0.8, 3.5) | 2.1  (0.8, 3.5) | 1.9  (0.3, 3.4) |
| DBP | | | | | |
| All  (n = 9479) | 84  (83, 84) | 81  (81, 81) | 2.9  (2.5, 3.3) | 3.0  (2.6, 3.4) | 3.0  (2.5, 3.4) |
| Pre-/peri-menopause  (n = 4743) | 85  (84, 85) | 80  (80, 81) | 4.5  (3.7, 5.3) | 4.3  (3.5, 5.1) | 4.0  (3.1, 4.8) |
| Early menopause  (n = 4670) | 85  (84, 85) | 81  (81, 82) | 3.8  (3.2, 4.4) | 3.7  (3.1, 4.4) | 3.6  (2.9, 4.2) |
| Late menopause  (n = 4095) | 82  (82, 83) | 81  (81, 82) | 1.2  (0.5, 1.9) | 1.6  (0.9, 2.3) | 2.0  (1.1, 2.8) |

Hypertension prevalence, mean systolic blood pressure (SBP), and mean diastolic blood pressure (DBP) are compared between women stratified by self-reported menopausal status and age-weighted men. Sex differences in hypertension are presented as prevalence ratios (PRs) with 95% confidence intervals (CIs), estimated using Poisson regression with robust variance after inverse probability weighting to match the age distribution of men to that of each menopausal group in women. Sex differences in blood pressure levels were assessed using weighted linear regression. Model A was adjusted for age and education; Model B additionally for diabetes, chronic kidney disease, and dyslipidemia; and Model C additionally for smoking, physical activity, body mass index, sodium intake, and potassium intake. CI: confidence interval, PR: prevalence ratio.

**Supplementary Table S6.** **Blood pressure levels and hypertension prevalence in postmenopausal women stratified by hormone replacement therapy.**

| Outcome | No HRT  (n = 2792)  (95% CI) | HRT  (n = 1089)  (95% CI) | Age- and education-adjusted beta / PR (95% CI)  (HRT vs. no HRT) | Fully adjusted  beta / PR (95% CI)  (HRT vs. no HRT) |
| --- | --- | --- | --- | --- |
| All | | | | |
| SBP, mmHg | 138.8  (138.1, 139.6) | **139.9**  **(138.8, 141.1)** | -1.01 (-2.35, 0.33) | -0.67 (-1.99, 0.65) |
| DBP, mmHg | 81.1  (80.8, 81.5) | 80.9  (80.3, 81.5) | -0.10 (-0.79, 0.58) | -0.02 (-0.70, 0.66) |
| Hypertension prevalence, % | 63.5  (61.7, 65.3) | 67.8  (65.0, 70.5) | 0.99 (0.91, 1.08) | 1.02 (0.93, 1.11) |
| Early menopause | | | | |
|  | **No HRT**  **(n = 1381)**  **(95% CI)** | **HRT**  **(n = 364)**  **(95% CI)** | **Age- and education-adjusted beta / PR (95% CI)**  **(per year)**  **(HRT vs. no HRT)** | **Fully adjusted**  **beta / PR (95% CI)**  **(per year)**  **(HRT vs. no HRT)** |
| SBP, mmHg | 133.1  (132.1, 134.0) | 133.3  (131.6, 135.1) | -0.10 (-2.14, 1.92) | 0.34 (-1.65, 2.34) |
| DBP, mmHg | 81.1  (80.6, 81.6) | 81.0  (80.1, 81.9) | -0.06 (-1.15, 1.04) | 0.17 (-0.91, 1.25) |
| Hypertension prevalence, % | 51.0 (48.4, 53.7) | 52.7 (47.6, 57.9) | 1.00 (0.85, 1.17) | 1.05 (0.89, 1.23) |
| Late menopause | | | | |
|  | **No HRT**  **(n = 1411)**  **(95% CI)** | **HRT**  **(n = 725)**  **(95% CI)** | **Age- and education-adjusted beta / PR (95% CI)**  **(per year)**  **(HRT vs. no HRT)** | **Fully adjusted**  **beta / PR (95% CI)**  **(per year)**  **(HRT vs. no HRT)** |
| SBP, mmHg | 144.5  (143.5, 145.6) | 143.3  (141.8, 144.7) | -1.53 (-3.32, 0.27) | -1.29 (-3.04, 0.47) |
| DBP, mmHg | 81.1  (80.6, 81.6) | 80.9  (80.2, 81.6) | -0.14 (-1.03, 0.13) | -0.10 (-0.35, 0.75) |
| Hypertension prevalence, % | 75.7  (73.5, 77.9) | 75.3  (72.2, 78.4) | 0.99 (0.89, 1.09) | 1.00 (0.90, 1.11) |

Values for systolic blood pressure (SBP) and diastolic blood pressure (DBP) are mean with standard deviation (SD). SBP and DBP were compared between women with and without hormone replacement therapy (HRT) using linear regression models estimating betas with 95% confidence intervals (CIs). Hypertension prevalence was compared using modified Poisson regression with robust standard errors estimating prevalence ratios (PRs) with 95% CIs. Models were adjusted for age and education or in the fully adjusted models for age, education, diabetes, dyslipidemia, chronic kidney disease, smoking status, physical activity, body mass index, sodium intake, and potassium intake. CI: confidence interval; DBP: diastolic blood pressure; HRT: hormone replacement therapy; PR: prevalence ratio; SBP: systolic blood pressure.

**Supplementary Table S7. Sex differences in awareness, treatment, and control across age-based life-stages.**

|  | **Prevalence men**  **(95% CI)** | **Prevalence women**  **(95% CI)** | **Age- and education-adjusted PR (95% CI)**  **(men vs women)** |
| --- | --- | --- | --- |
| **Awareness** | | | |
| All (n = 6382) | 0.58 (0.56, 0.59) | 0.59 (0.57, 0.61) | 1.01 (0.96, 1.05) |
| 45–54 years (n =920) | 0.42 (0.38, 0.46) | 0.46 (0.41, 0.51) | 0.97 (0.84, 1.20) |
| 55–64 years (n = 1974) | 0.58 (0.55, 0.61) | 0.59 (0.55, 0.62) | 1.02 (0.94, 1.01) |
| 65–74 years (n = 3488) | 0.62 (0.59, 0.64) | 0.63 (0.61, 0.65) | 1.00 (0.94, 1.05) |
| **Treatment** | | | |
| All (n = 6382) | 0.58 (0.57, 0.60) | 0.60 (0.58, 0.62) | 1.01 (0.97, 1.05) |
| 45–54 years (n =920) | 0.36 (0.32, 0.40) | 0.44 (0.39, 0.49) | 0.85 (0.73, 1.00) |
| 55–64 years (n = 1974) | 0.56 (0.53, 0.59) | 0.58 (0.55, 0.61) | 0.98 (0.91, 1.06) |
| 65–74 years (n = 3488) | 0.66 (0.64, 0.68) | 0.64 (0.62, 0.67) | 1.03 (0.98, 1.09) |
| C**ontrol** | | | |
| All (n = 3767) | 0.40 (0.38, 0.42) | 0.45 (0.42, 0.47) | 0.91 (0.85, 0.99) |
| 45–54 years (n = 364) | 0.45 (0.38, 0.52) | 0.52 (0.44, 0.59) | 0.89 (0.73, 1.10) |
| 55–64 years (n = 1121) | 0.43 (0.39, 0.47) | 0.56 (0.51, 0.60) | 0.78 (0.69, 0.88) |
| 65–74 years (n = 2282) | 0.38 (0.35, 0.41) | 0.38 (0.35, 0.41) | 1.03 (0.92, 1.15) |

Hypertension awareness and treatment were analyzed among all individuals with hypertension. Hypertension control was analyzed in hypertensive individuals receiving blood pressure lowering medication. Analyses were stratified by age-based life-stages. Sex-differences, given as prevalence ratios (PRs) with 95% confidence intervals (CIs) between men and women, were analyzed by Poisson regression models adjusted for age and education with robust variance estimation. CI: confidence interval; PR: prevalence ratio.

**Supplementary Table S8. Sex differences in antihypertensive drug treatment.**

|  | **Women**  **(n = 2811)** | **Men**  **(n = 3318)** | **Model A**  **PR**  **(95% CI)** | **p-value** |
| --- | --- | --- | --- | --- |
| Number of antihypertensive medications, median (IQR) | 1 (0, 2) | 1 (0, 2) | 1.1  (1.04–1.16) | <0.001 |
| ACE-inhibitors, n (%) | 572 (20.3) | 831 (25) | 1.33  (1.21–1.46) | < 0.001 |
| Angiotensin-receptor-blockers, n (%) | 687 (24.4) | 790 (23.8) | 0.95  (0.86–1.04) | 0.259 |
| Calcium channel blockers, n (%) | 380 (13.5) | 540 (16.3) | 1.27  (1.12–1.44) | < 0.001 |
| Thiazide diuretics, n (%) | 548 (19.5) | 656 (19.8) | 1.06  (0.95–1.17) | 0.287 |
| Betablockers, n (%) | 780 (27.7) | 881 (26.6) | 1.01  (0.93–1.10) | 0.813 |
| Loop diuretics, n (%) | 96 (3.4) | 108 (3.3) | 1.08  (0.82–1.42) | 0.596 |
| Mineralocorticoid receptor antagonists, n (%) | 25 (0.9) | 44 (1.3) | 1.67  (1.01–2.74) | 0.044 |

Sex differences in antihypertensive drug usage were investigated among all participants with prevalent hypertension and available data for medication use (n = 6,129). Prevalence ratios (PR) and 95% confidence intervals (CI) comparing men to women were estimated using Poisson regression. Model A was adjusted for age and education. PR: prevalence ratio; CI: confidence interval; IQR: interquartile range; ACE: angiotensin-converting enzyme.
